# Supplementary material for: Efficient and stable catalytic hydrolysis of perfluorocarbon enabled by SO2-mediated proton supply
Source: Nat Commun. 2026 Jan 14;17:597. doi: 10.1038/s41467-026-68386-4 (PMC12808091; doi:10.1038/s41467-026-68386-4)
Supplement: Supplementary file 1 — Supplementary Information [file 41467_2026_68386_MOESM1_ESM.pdf]

Supplementary Information

Efficient and stable catalytic hydrolysis of perfluorocarbon enabled by SO_2-mediated proton supply

Hang Zhang[1,2], Tao Luo[1,3], Yingkang Chen[1], Xiaojian Wang[1], Edoardo Mariani[2], Kang Liu[1], Junwei Fu[1], Changxu Liu[4], Hui Liu[5], Zhang Lin[5], Liyuan Chai[5], Michelle L. Coote[3], Emiliano Cortés[2,*] and Min Liu[1,5,*]

[1] Hunan Joint International Research Center for Carbon Dioxide Resource Utilization, School of Physics, Central South University, Changsha 410083, Hunan, P.R. China; E-mail: minliu@csu.edu.cn

[2] Nanoinstitut München, Fakultät für Physik, Ludwig-Maximilians-Universität München, 80539 München, Germany; E-mail: Emiliano.Cortes@lmu.de

[3] Institute for Nanoscale Science & Technology, Flinders University, Bedford Park, South Australia 5042, Australia.

[4] Centre for Metamaterial Research & Innovation, Department of Engineering, University of Exeter, Exeter EX4 4QF, United Kingdom;

[5] School of Metallurgy and Environment, Central South University, Changsha 410083, Hunan, P.R. China;

Additional Figures

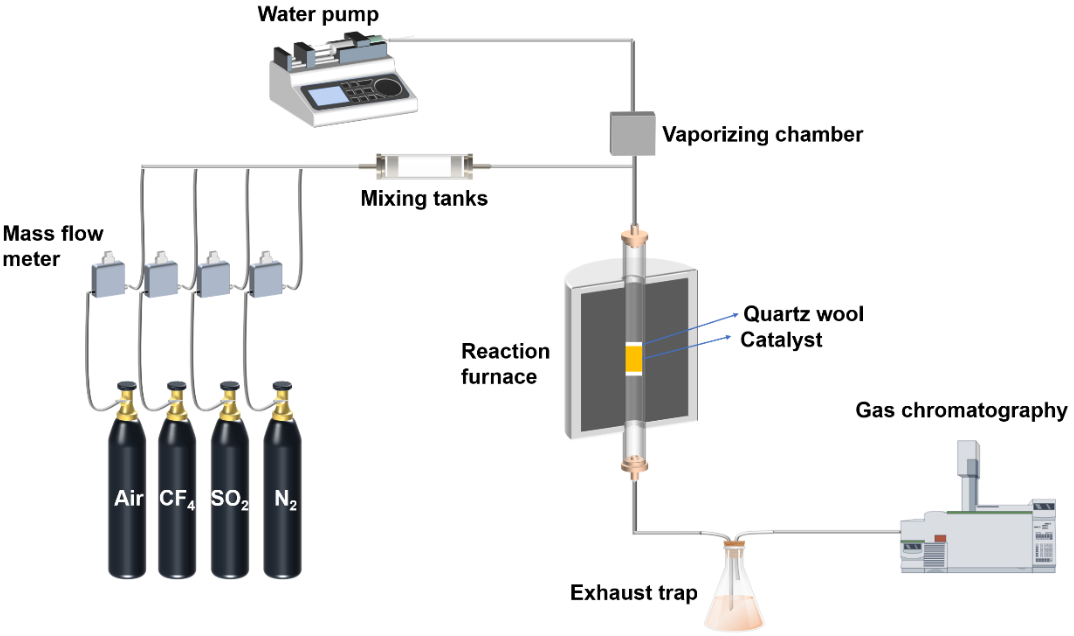

Supplementary Fig. 1 | Schematic diagram of the SO_2 and CF_4 removal system.

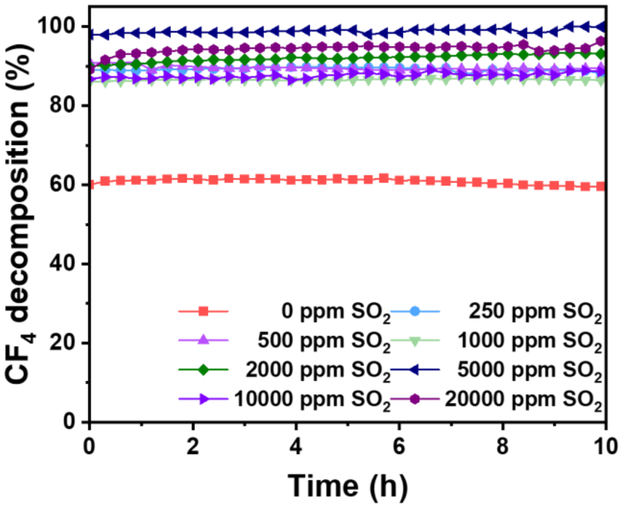

Supplementary Fig. 2 | CF_4 decomposition (%) during the CF_4 and SO_2 synergistic reaction at different SO_2 concentration (250-20000 ppm).

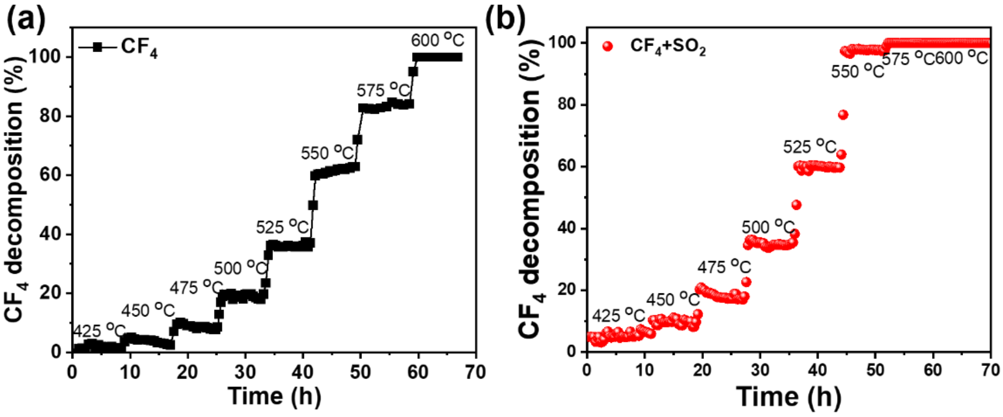

Supplementary Fig. 3 | Catalytic performance under different reaction temperatures. (a) CF_4 decomposition (%) during CF_4 catalytic hydrolysis reactions at different reaction temperatures. (b) CF_4 decomposition (%) during the CF_4 and SO_2 synergistic reaction at different reaction temperatures.

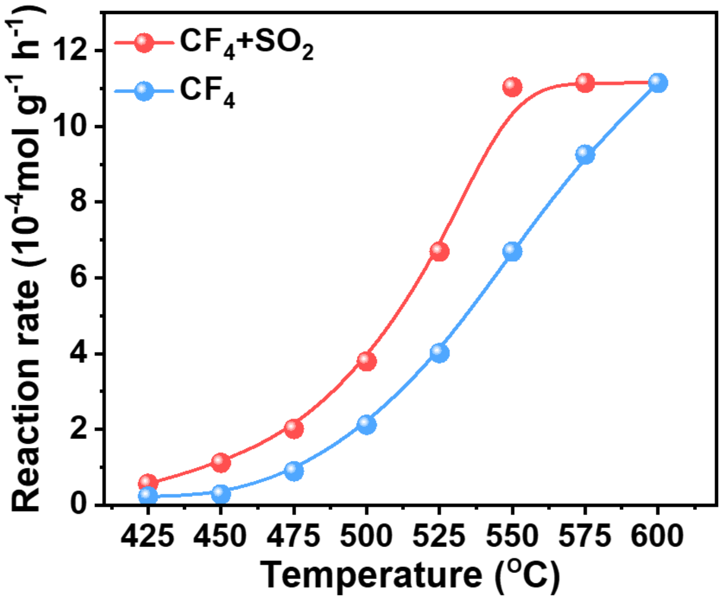

Supplementary Fig. 4 | Reaction rate (CF_4) of CF_4 catalytic hydrolysis reactions and CF_4 and SO_2 synergistic reaction at different reaction temperatures.

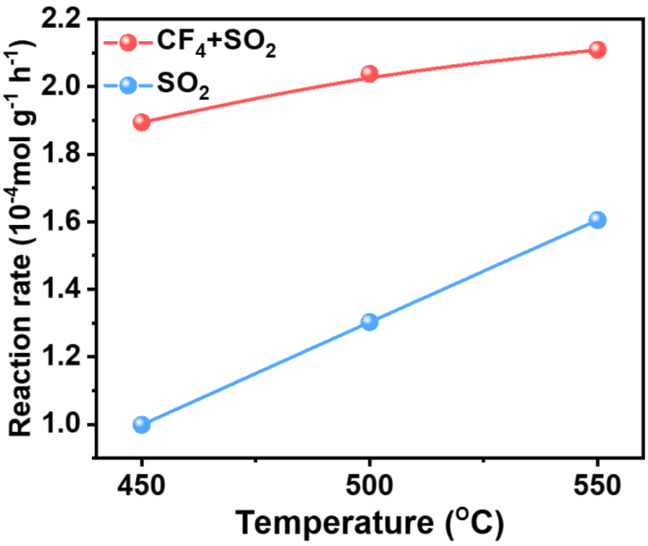

Supplementary Fig. 5 | Reaction rate (SO_2) of SO_2 catalytic oxidation reactions and CF_4 and SO_2 synergistic reaction at different reaction temperatures.

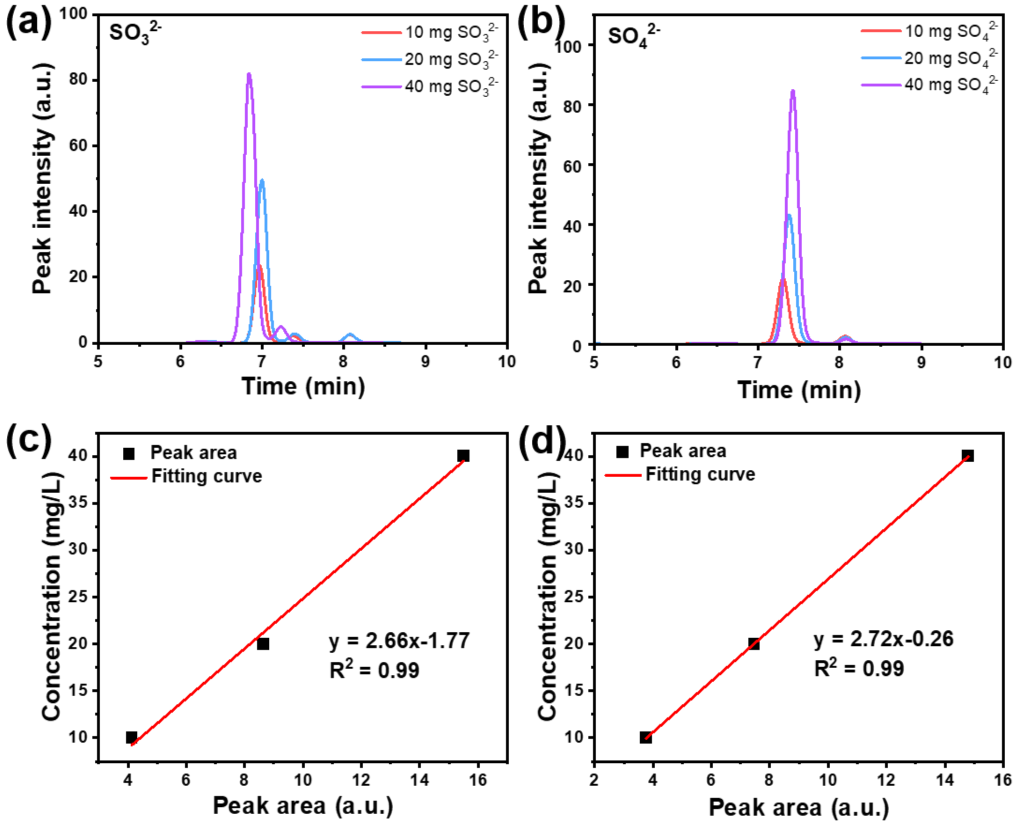

Supplementary Fig. 6 | The SO_4^{2-} and SO_3^{2-} content standard curve of Ion chromatography (IC). (a) The IC spectra of standard SO_3^{2-} sample. (b) The IC spectra of standard SO_4^{2-} sample. (c) The linear fitting of SO_3^{2-} content with peak area. (d) linear fitting of SO_4^{2-} content with peak area.

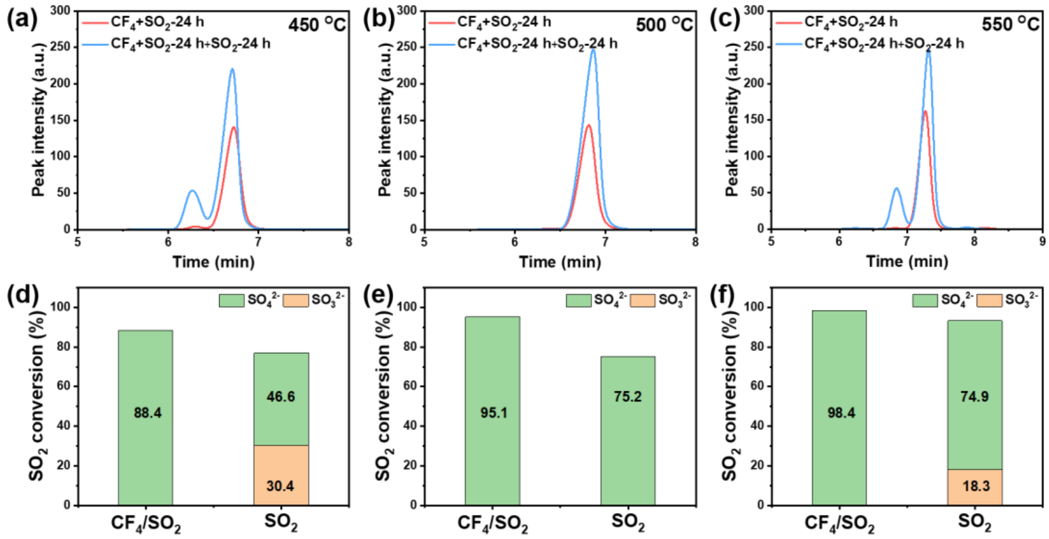

Supplementary Fig. 7 | Evaluation of SO_2 catalytic oxidation performance. The SO_4^{2-} and SO_3^{2-} content of SO_2 catalytic oxidation reactions and CF_4/SO_2 synergistic reaction at different reaction temperatures (a) 450 ℃, (b) 500 ℃ and (c) 550 ℃. The SO_2 conversion (%) of SO_2 catalytic oxidation reactions and CF_4/SO_2 synergistic reaction at different reaction temperatures (d) 450 ℃, (e) 500 ℃ and (f) 550 ℃.

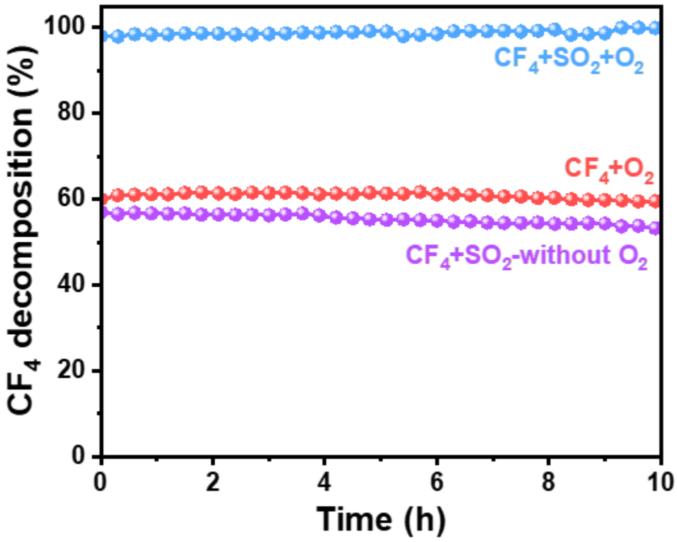

Supplementary Fig. 8 | CF_4 decomposition (%) under different condition.

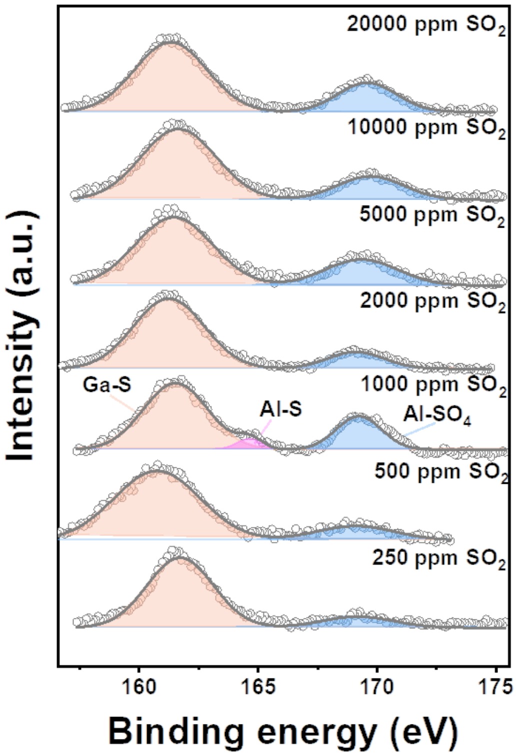

Supplementary Fig. 9 | XPS spectra of S 2p for the used samples during the CF_4 and SO_2 synergistic reaction at different SO_2 concentration (250-20000 ppm).

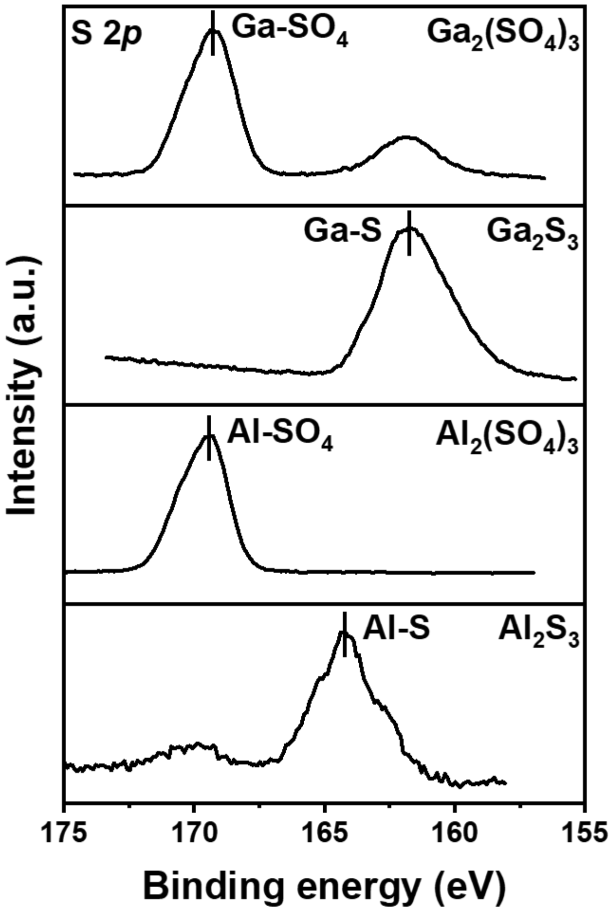

Supplementary Fig. 10 | XPS spectra of S 2p for Al_2S_3, $Al_2(SO_4)_3$, Ga_2S_3 and $Al_2(SO_4)_3$.

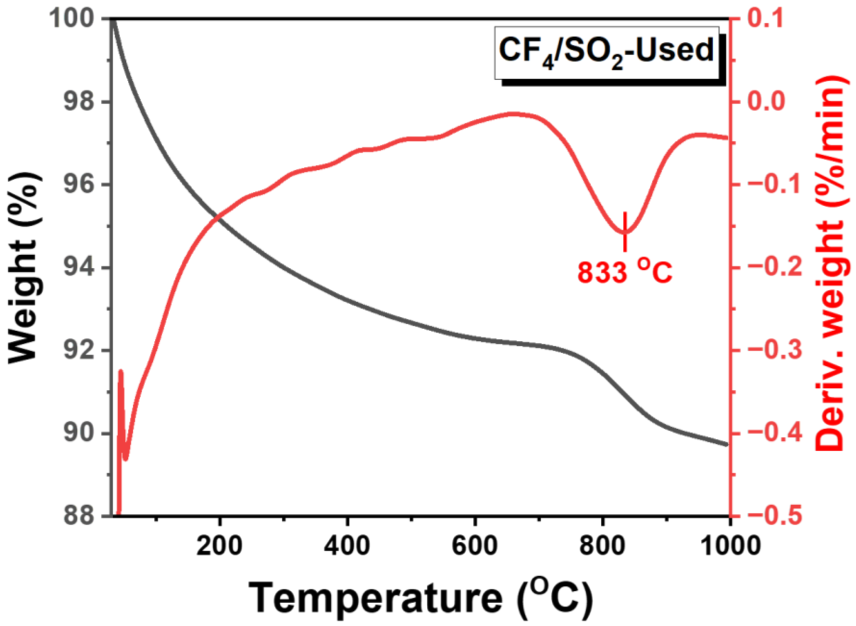

Supplementary Fig. 11 | TG curve of the CF_4/SO_2-used sample.

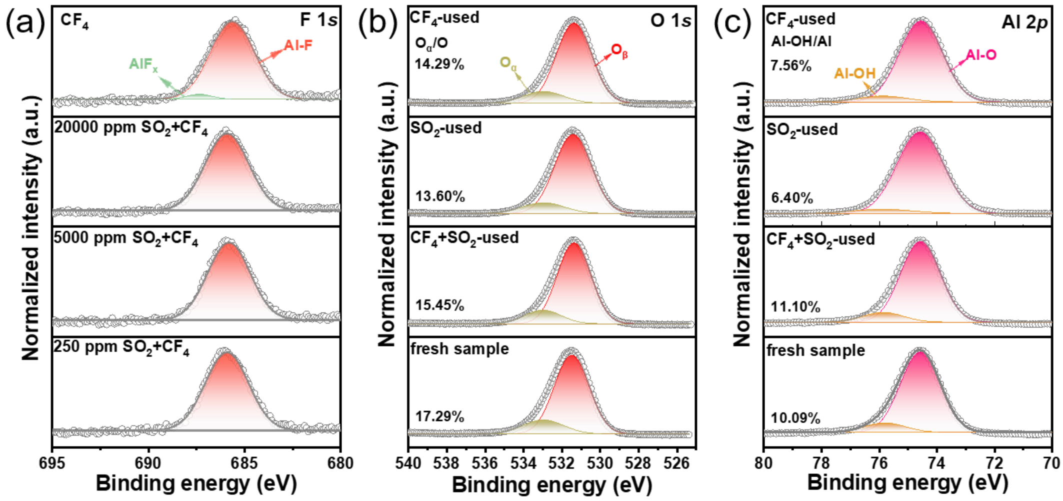

Supplementary Fig. 12 | XPS characterization. XPS spectra of (a) F 1s, (b) O 1s and (c) Al 2p for the samples.

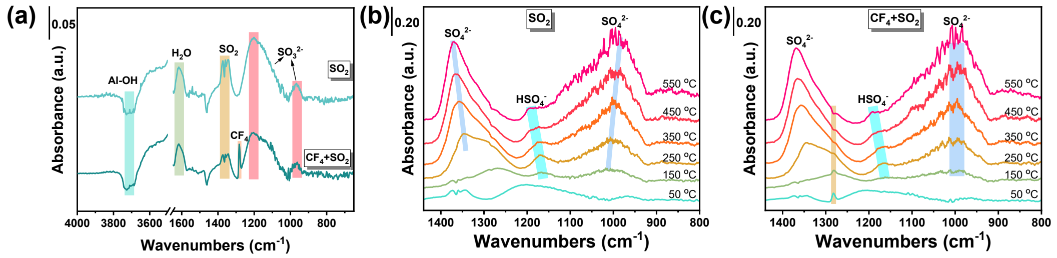

Supplementary Fig. 13 | In situ DRIFTS measurements. (a) In situ DRIFTS measurements of the preadsorption of SO_2 solo, SO_2 and CF_4 over Ga/θ-Al_2O_3 catalyst, respectively. (b) In situ DRIFTS measurements of following SO_2 preadsorption as a function of temperature. (c) In situ DRIFTS measurements of following SO_2 and CF_4 preadsorption as a function of temperature.

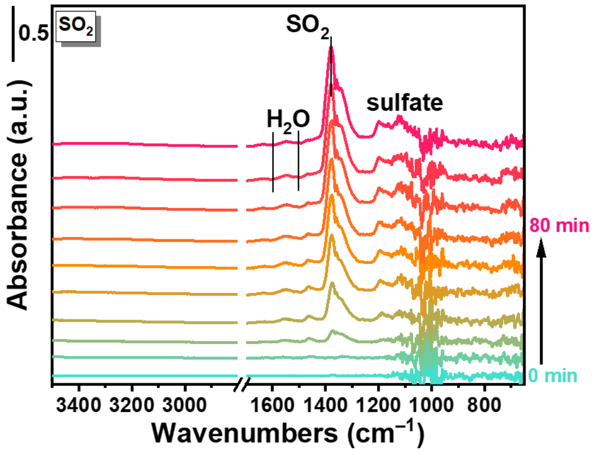

Supplementary Fig. 14 | In situ DRIFTS of SO_2 catalytic oxidation over Ga/θ-Al_2O_3 catalyst under 550 °C with a function as time.

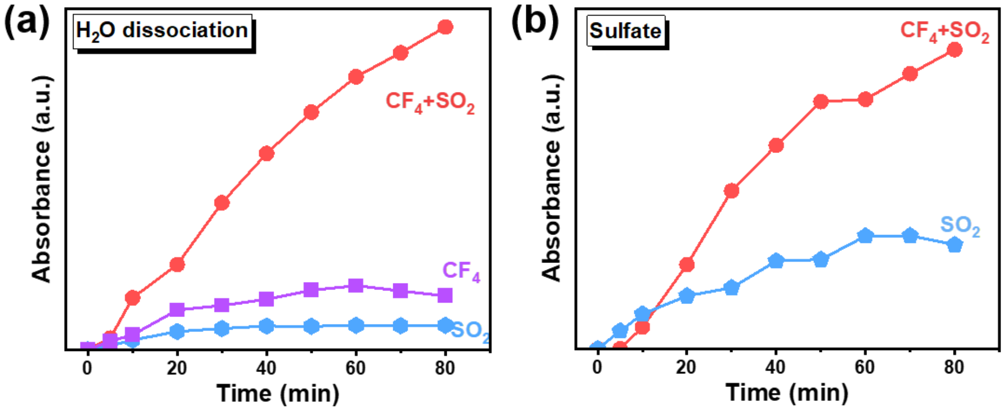

Supplementary Fig. 15 | The absorbance of (a) H_2O dissociation and (b) sulfate species with a function as time.

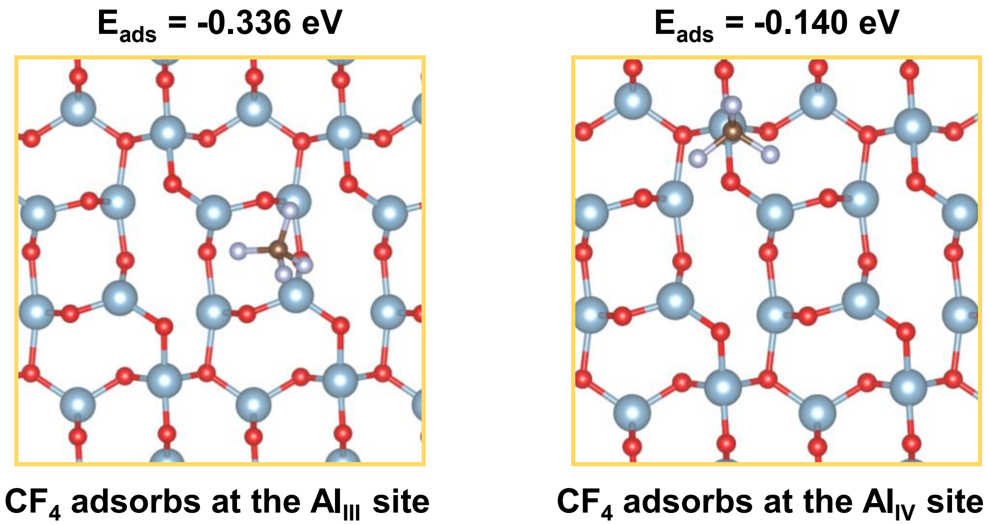

Supplementary Fig. 16 | Adsorption energy of CF_4 adsorbed on Al_{III} and Al_{IV} sites on the exposed θ-Al_2O_3 (010) surface.

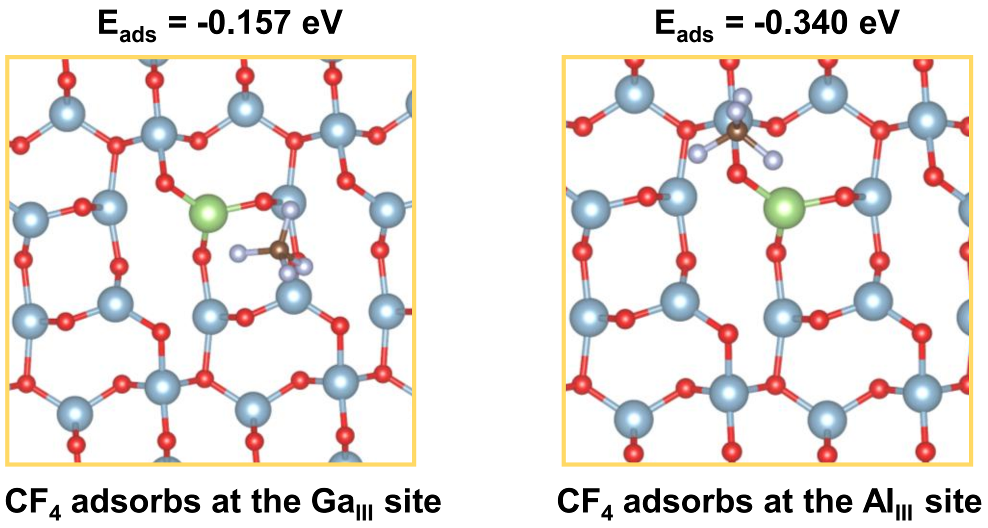

Supplementary Fig. 17 | Adsorption energy of CF_4 adsorbed on Ga_{III} and Al_{III} sites on the exposed Ga/θ-Al_2O_3 (010) surface.

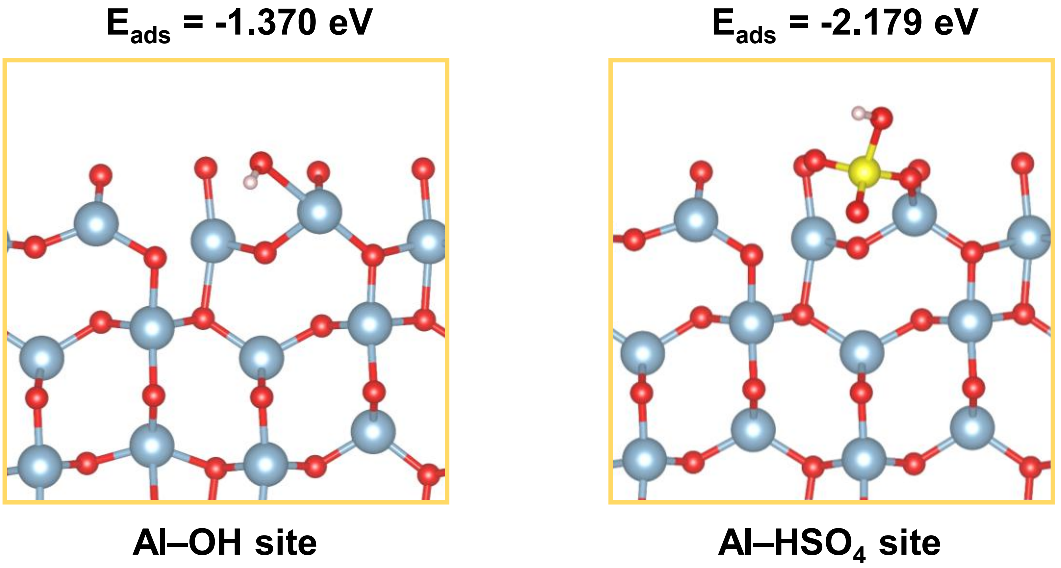

Supplementary Fig. 18 | The adsorption energies of $–HSO_4$ and $–OH$ adsorbed on the exposed θ-Al_2O_3 (010) surface.

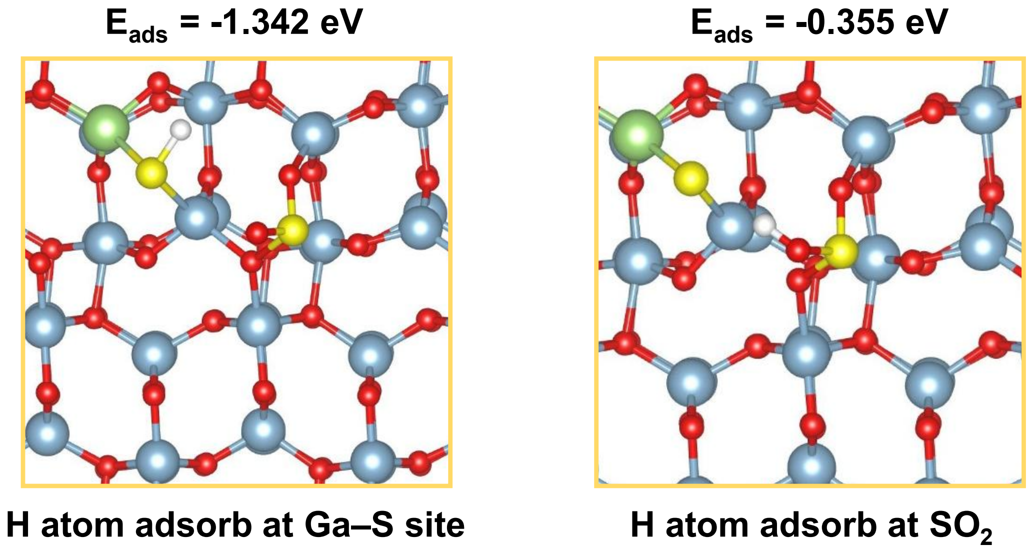

Supplementary Fig. 19 | The adsorption energies of H atom adsorbed at Ga–S site and SO_2, respectively.

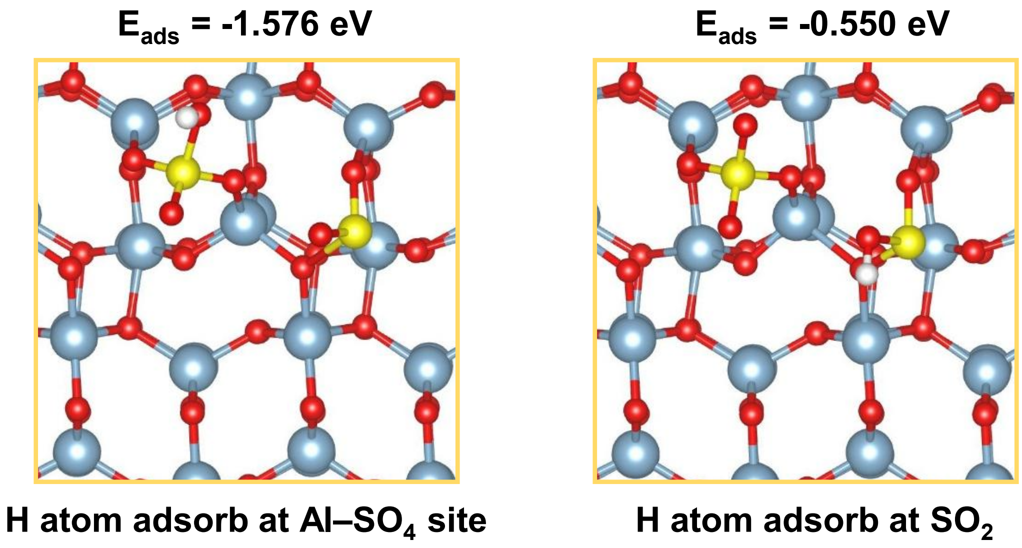

Supplementary Fig. 20 | The adsorption energies of H atom adsorbed at Al–HSO_4 site and SO_2, respectively.

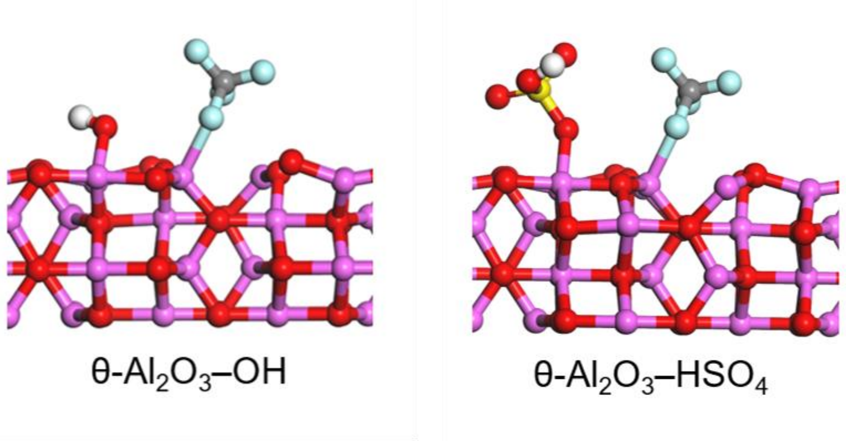

Supplementary Fig. 21 | The calculation model of CF_4 adsorption on θ-Al_2O_3–OH and θ-Al_2O_3–HSO_4.

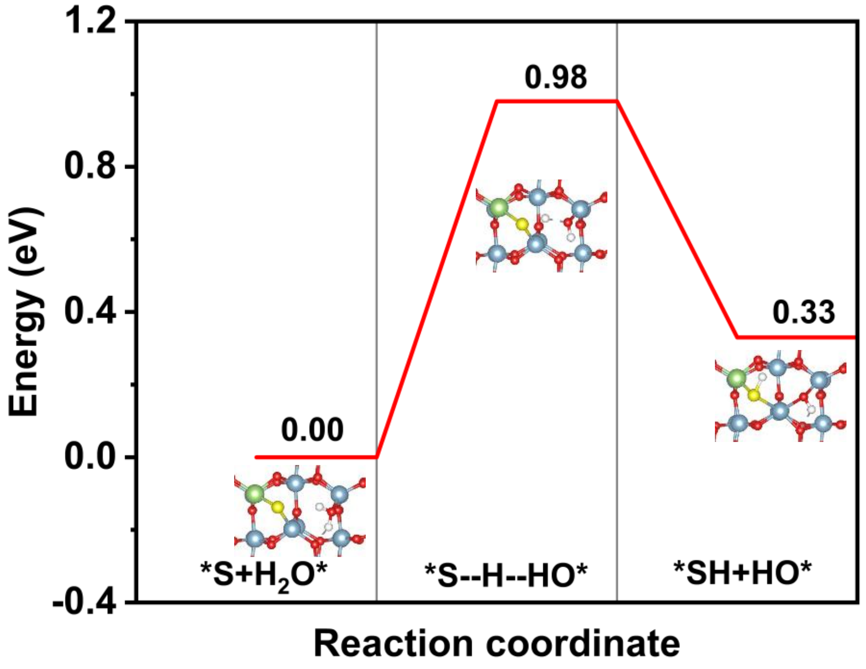

Supplementary Fig. 22 | Reaction energy profiles and related structures of the Ga–HS structure regeneration.

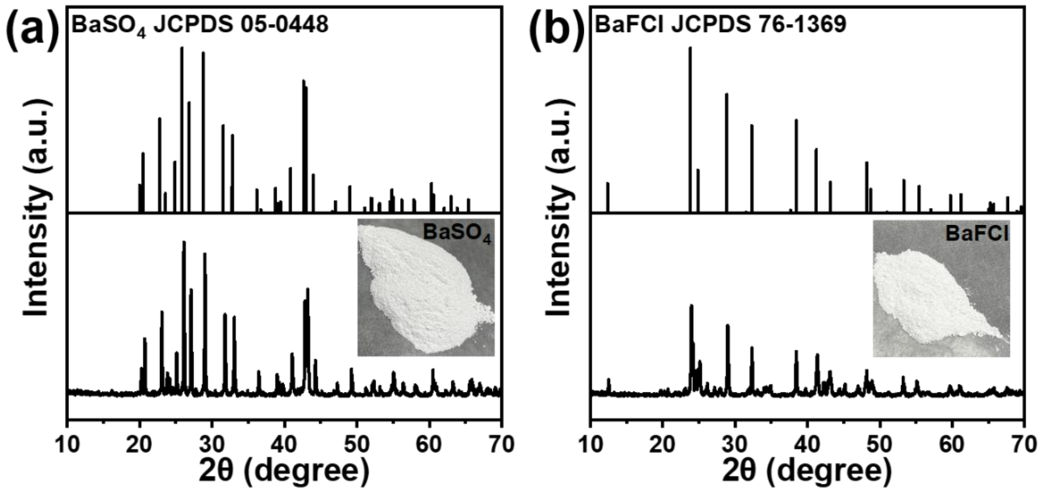

Supplementary Fig. 23 | Resource recycling of products. (a) XRD pattern of the recovered $BaSO_4$. (b) XRD pattern of the recovered BaFCl.

Supplementary Table 1 | The molar of SO_4^{2-} and SO_2 conversion for CF_4 and SO_2 synergistic elimination stability tests

**Time (hours)**	**Molar of SO_4^{2-} （mol）**	**SO_2 Conversion (%)**
24	0.104584988	97.71037
48	0.105632311	98.68885
72	0.105360002	98.43444
96	0.105380949	98.45401
120	0.10517149	98.25832
144	0.105527577	98.591
168	0.10370524	96.88845
192	0.103956603	97.12329
216	0.105318108	98.3953
240	0.105318108	98.3953
264	0.10437553	97.51468
288	0.103370101	96.57534
312	0.103663357	96.84932
336	0.103684293	96.86888
360	0.10370524	96.88845
384	0.10477351	97.8865
408	0.105066756	98.16047
432	0.104647829	97.76908
456	0.104270796	97.41683
480	0.10370524	96.88845
504	0.103391048	96.59491
528	0.103809975	96.9863
552	0.104333636	97.47554
576	0.104459307	97.59295
600	0.104438371	97.57339
624	0.104438371	97.57339
648	0.104522148	97.65166
672	0.104647829	97.76908
696	0.104857287	97.96477
720	0.104689723	97.80822
744	0.104857287	97.96477
768	0.104899181	98.00391
792	0.104689723	97.80822
816	0.104794457	97.90607
840	0.104605935	97.72994
864	0.104396477	97.53425
888	0.103914709	97.08415
912	0.104501201	97.63209
936	0.10437553	97.51468
960	0.103391048	96.59491

984	0.103663357	96.84932
1008	0.10284645	96.08611
1032	0.103577386	96.769
1056	0.103844975	97.019
1080	0.104400491	97.538
1104	0.104524652	97.654
1128	0.104462572	97.596
1152	0.104586733	97.712
1176	0.103474632	96.673
1200	0.103330134	96.538
1224	0.10361913	96.808
1248	0.104071891	97.231
1272	0.10433948	97.481
1296	0.104482908	97.615
1320	0.103927393	97.096
1344	0.104215319	97.365
1368	0.104524652	97.654
1392	0.102857036	96.096
1416	0.103248787	96.462
1440	0.103227379	96.442
1464	0.102857036	96.096
1488	0.10433948	97.481
1512	0.102857036	96.096
1536	0.104812578	97.923
1560	0.104750498	97.865
1584	0.105409838	98.481
1608	0.103001534	96.231
1632	0.103639467	96.827
1656	0.103494969	96.692
1680	0.104319144	97.462
1704	0.104421898	97.558
1728	0.104051554	97.212
1752	0.104051554	97.212
1776	0.104154309	97.308
1800	0.103124625	96.346
1824	0.104894996	98
1848	0.104977413	98.077
1872	0.105182922	98.269
1896	0.103742221	96.923
1920	0.10466808	97.788
1944	0.104544989	97.673
1968	0.103907056	97.077
1992	0.104482908	97.615

2016	0.104092228	97.25
2040	0.102857036	96.09663744
2064	0.103474632	96.67364127
2088	0.10328946	96.50064012
2112	0.103351541	96.5586405
2136	0.102981197	96.21263821
2160	0.102981197	96.21263821
2184	0.103083952	96.30863884
2208	0.102054268	95.34663246
2232	0.103824639	97.00064343
2256	0.103907056	97.07764395
2280	0.104112565	97.26964522
2304	0.102671864	95.92363629
2328	0.103597723	96.78864203
2352	0.103474632	96.67364127
2376	0.102836699	96.07763731
2400	0.103412551	96.61564088
2424	0.103021871	96.25063846
2448	0.102857036	96.09663744
2472	0.103412551	96.61564088
2496	0.103969137	97.13564433
2520	0.103844975	97.01964356

Supplementary Table 2 | Comprehensive comparison of the various activity parameters with reported results for CF_4 catalytic hydrolysis.

Catalysts	Decomposition (%)	Reaction temperatures (°C)	Lifetime (hour)	Ref.
**Ga/θ-Al_2O_3**	**100**	**550**	**2,500**	**This work**
Zn-Al_2O_3	100	560	250	Luo, W. et al.[1]
γ-Al_2O_3	100	600	60	Luo, T. et al.[2]
S-Al_2O_3@ZrO_2	100	580	10	Chen, Y. et al.[3]
Ga/θ-Al_2O_3	100	600	1,000	Zhang, H. et al.[4]
Ni/Al_2O_3	100	570	300	Wang, X. et al.[5]
γ-Al_2O_3	100	650	3	Zhang, H. et al.[6]
yolk–shell Al_2O_3	100	580	160	Zheng, J. et al.[7]
S-$ZnAl_2O_4$	100	600	20	Wang, X. et al.[8]
S-Ce-HZSM-5	100	650	60	Zheng, X. et al.[9]
S/Ce/γ-Al_2O_3	50	650	45	Song, J.-Y. et al.[10]
SO_4^{2-}/Ga/γ-Al_2O_3	98	630	72	El-Bahy, Z. M. et al.[11]

Supplementary Table 3 | Adsorption energy of CF_4 on θ-Al_2O_3 and Ga/θ-Al_2O_3 surfaces.

**Catalyst**	**Site**	**CF_4 adsorption energy (eV)**
θ-Al_2O_3	Al_{III}	-0.336 eV
	Al_{IV}	-0.140 eV
Ga/θ-Al_2O_3	Ga_{III}	-0.157 eV
	Al_{III}	-0.340 eV

Supplementary Table 4 | The CF_4 adsorption energy at Al_{III} site for θ-Al_2O_3–OH and θ-Al_2O_3–HSO_4 model.

Model	CF_4 adsorption energy
θ-Al_2O_3–OH	-0.15 eV
θ-Al_2O_3–HSO_4	-0.50 eV

Supplementary Table 5 | Typical electrolytic aluminum flue gas composition.

	**Fluoride mg/m^3**	**SO_2 mg/m^3**	**CF_4 (ppm)**	**N_2 (%)**	**O_2 (%)**	**CO (%)**	**CO_2 (%)**
**Concentration**	40-50	150-380	1500-2000 ppm	78	20.3-20.6	0.05-0.15	0.9-1.5

Supplementary References

1. Luo, W. *et al.* Promoting C–F bond activation for perfluorinated compounds decomposition via atomically synergistic lewis and brønsted acid sites. *J. Am. Chem. Soc.* **147**, 7391–7399 (2025).
2. Luo, T. *et al.* Unveiling tetrafluoromethane decomposition over alumina catalysts. *J. Am. Chem. Soc.* **146**, 35057–35063 (2024).
3. Chen, Y. *et al.* Promoting C–F bond activation via proton donor for CF_4 decomposition. *Proc. Natl. Acad. Sci.* **120**, e2312480120 (2023).
4. Zhang, H. *et al.* Highly efficient decomposition of perfluorocarbons for over 1000 hours via active site regeneration. *Angew. Chem. Int. Ed.* **62**, e202305651 (2023).
5. Wang, X. *et al.* Detoxification of carbonaceous species for efficient perfluorocarbon hydrolysis. *Environ. Sci. Technol.* **59**, 3309–3315 (2025).
6. Zhang, H. *et al.* Identification of the active site during CF_4 hydrolytic decomposition over γ-Al_2O_3. *Environ. Sci.: Nano* **9**, 954–963 (2022).
7. Zheng, J. *et al.* Catalytic hydrolysis of perfluorinated compounds in a yolk–shell micro-reactor. *Adv. Sci.* 2413203 (2025) doi:10.1002/advs.202413203.
8. Wang, X. *et al.* Promoted CF4 decomposition via enhanced tricoordinated Al active sites. *ACS ES&T Engg.* **4**, 1142–1148 (2024).
9. Zheng, X., Chen, S., Liu, W., Xiang, K. & Liu, H. The design of sulfated Ce/HZSM-5 for catalytic decomposition of CF_4. *Polymers* **14**, 2717 (2022).
10. Song, J.-Y. *et al.* The catalytic decomposition of CF_4 over Ce/Al_2O_3 modified by a cerium sulfate precursor. *J. Mol. Catal. A: Chem.* **370**, 50–55 (2013).
11. El-Bahy, Z. M., Ohnishi, R. & Ichikawa, M. Hydrolytic decomposition of CF_4 over alumina-based binary metal oxide catalysts: high catalytic activity of gallia-alumina catalyst. *Catal. Today* **90**, 283–290 (2004).

Additional Computational Details

The INCAR for DFT calculation:

ISTART = 0
ICHARG = 2
INIWAV = 1
ISPIN = 2
ENCUT = 450
NELM = 120
NELMIN = 2
EDIFF = 1E-05
PREC = Normal
LREAL = Auto
EDIFFG = -0.01
ISIF = 2
ISMEAR = 0
SIGMA = 0.05
ALGO = Fast
IVDW= 11
LVDW = .TRUE.
IWAVPR = 11
NSW = 300
IBRION = 2

The INCAR for cAIMD simulations:

```
ISTART = 0
ICHARG = 2
INIWAV = 1
ISPIN = 1
ENCUT = 450
NELM = 120
NELMIN = 6
EDIFF = 1E-05
PREC = Normal
LREAL = Auto
EDIFFG = -0.05
ISIF = 2
ISMEAR = 0
SIGMA = 0.05
ALGO = V
IVDW= 11
LVDW = .TRUE.
# for cAIMD
IWAVPR = 11
NSW = 3000
IBRION = 0
POTIM = 1.0
NBLOCK = 1
KBLOCK= 100
TEBEG = 823
MDALGO = 2
SMASS = 0
INCREM = 0.001
LBLUEOUT = T
```
